# Why georeferencing matters: Introducing a practical protocol to prepare species occurrence records for spatial analysis

**DOI:** 10.1002/ece3.3516

**Published:** 2017-12-06

**Authors:** Trevor D. S. Bloom, Aquila Flower, Eric G. DeChaine

**Affiliations:** ^1^ Department of Biology Western Washington University Bellingham WA USA; ^2^ Department of Environmental Studies Western Washington University Bellingham WA USA

**Keywords:** biogeography, botany, georeferencing, GIS, herbarium records, museum collections, Species Distribution Models

## Abstract

Species Distribution Models (SDMs) are widely used to understand environmental controls on species’ ranges and to forecast species range shifts in response to climatic changes. The quality of input data is crucial determinant of the model's accuracy. While museum records can be useful sources of presence data for many species, they do not always include accurate geographic coordinates. Therefore, actual locations must be verified through the process of georeferencing. We present a practical, standardized manual georeferencing method (the Spatial Analysis Georeferencing Accuracy (SAGA) protocol) to classify the spatial resolution of museum records specifically for building improved SDMs. We used the high‐elevation plant *Saxifraga austromontana* Wiegand (Saxifragaceae) as a case study to test the effect of using this protocol when developing an SDM. In MAXENT, we generated and compared SDMs using a comprehensive occurrence dataset that had undergone three different levels of georeferencing: (1) trained using all publicly available herbarium records of the species, minus outliers (2) trained using herbarium records claimed to be previously georeferenced, and (3) trained using herbarium records that we have manually georeferenced to a ≤ 1‐km resolution using the SAGA protocol. Model predictions of suitable habitat for *S. austromontana* differed greatly depending on georeferencing level. The SDMs fitted with presence locations georeferenced using SAGA outperformed all others. Differences among models were exacerbated for future distribution predictions. Under rapid climate change, accurately forecasting the response of species becomes increasingly important. Failure to georeference location data and cull inaccurate samples leads to erroneous model output, limiting the utility of spatial analyses. We present a simple, standardized georeferencing method to be adopted by curators, ecologists, and modelers to improve the geographic accuracy of museum records and SDM predictions.

## 1 INTRODUCTION

Climate change is predicted to result in massive species range shifts and population‐level extinctions (Clark, Bell, Kwit, & Zhu, 2014; Hijmans & Graham, 2006; Thomas et al., 2004; Thuiller, Lavorel, Araújo, Sykes, & Prentice, 2005). Observing, describing, and forecasting patterns of biodiversity under changing climate conditions are critical goals in the fields of biogeography, conservation, and ecology (Bucklin et al., 2015). Species Distribution Models (SDMs), also referred to as Bioclimatic Envelope Models, are the most widely used approach for predicting past, present, and future suitable habitats for common and rare species (Elith, Kearney, & Phillips, 2010; Hijmans & Graham, 2006; Phillips & Dudík, 2008; Wiens, Stralberg, Jongsomjit, Howell, & Snyder, 2009). These models are used to predict climate change impacts (Keith et al., 2008; Serra‐Diaz et al., 2014; Wiens et al., 2009), construct phylogeographic patterns (Forester, DeChaine, & Bunn, 2013), and guide efforts to locate new populations of rare species (Williams et al., 2009). Reliable SDMs can inform land managers where to concentrate conservation resources to best preserve areas of ecological importance. Because SDMs rely on species occurrence coordinates, climate data, and other environmental variables to define a species’ bioclimatic niche and project future ranges (Bucklin et al., 2015; Flower, Murdock, Taylor, & Zwiers, 2013), the accuracy of those variables strongly affects the reliability of the model's predictions. In this paper, we analyze the effects of using species presence records of varying accuracy, demonstrating the importance of rigorous georeferencing to obtain optimal SDM results.

Although there are a variety of modeling methods and algorithms for generating SDMs, correlative models constructed using only species occurrence records and climate data are commonly used tools (Bucklin et al., 2015; Flower et al., 2013; Guillera‐Arroita et al., 2015; Oke & Thompson, 2015). These models do not include true absence data, nor do they explicitly account for additional variables such as interspecies interactions or species’ dispersal abilities (Flower et al., 2013; Pearson & Dawson, 2003). Correlative models predict the realized niche of the species, not the fundamental niche, due to their reliance on observed presence records (Wiens et al., 2009). There are several notable sources of uncertainty in the process of SDM development (Wiens et al., 2009). One source of uncertainty arises because of the fact that any ecological or climatic model is constrained by the selection of environmental variables. While there is no consensus as to which environmental or climate variables are to be included in standard SDMs, many agree that the selection of variables can potentially introduce bias (Bucklin et al., 2015). A model's accuracy is also constrained by the resolution and quality of the climate data (Real, Luz Márquez, Olivero, & Estrada, 2010). Climate data are usually represented as continuous grids interpolated from quality‐controlled climate station datasets (Daly et al., 2008). The quality of these climate data and the methods of interpolating from point records to a continuous surface and correcting for factors such as elevation and aspect can be sources of error in SDMs (Real et al., 2010). There can also be issues regarding the taxonomic identification of the specimen (Lozier, Aniello, & Hickerson, 2009). Species can be misidentified, or the systematics and taxonomy may have evolved over the years to include different species classifications. Sampling bias and imperfect detection are also noted limitations of the current available data for species distributions (Boakes et al., 2010; Fourcade, Engler, Rödder, & Secondi, 2014; Guillera‐Arroita et al., 2015; Newbold, 2010). Among all these potential sources of model uncertainty, one particularly important variable for creating reliable SDMs is the accuracy of the species occurrence localities (Newbold, 2010).

Museum and herbarium records can provide valuable information on the distribution of extinct and extant species (Anderson, 2012; Davis, Willis, Connolly, Kelly, & Ellison, 2015; Newbold, 2010). Millions of occurrence records can be accessed directly from the museum or in reputable online databases, many publicly available (Newbold, 2010). Most include a written site description and often geographic coordinates (see Fig. S1 in Supporting Information). The quality of location data generally declines with specimen age. Herbarium records’ site descriptions and associated geographic coordinates are frequently used to build high‐resolution SDMs (Alvarado‐Serrano & Knowles, 2014; Forester et al., 2013; Lozier et al., 2009). Site coordinates should have as good or better resolution than the climate data, often ≤1 km^2^, in order to produce useful SDMs (Wiens et al., 2009). Failure to assess spatial error in these occurrence record coordinates can have significant impacts on apparent species distributions (Rowe, 2005), although the severity of this effect varies among species and is partially dependent on the modeling method used (Graham et al., 2008). Several studies address the effect of sampling bias on SDM output (Boakes et al., 2010; Fourcade et al., 2014; Phillips et al., 2009), but less attention has been paid to the standardization of georeferencing to improve model performance. Previous research on the role of locational accuracy has focused on the effects of adding simulated random locational error (Graham et al., 2008), rather than assessing the error in actual museum records.

Most herbarium and museum records were not documented by collectors with the intention of use in geographic modeling, resulting in many potential sources of spatial error (Bowe & Haq, 2010). Recently, there have been increasing inventories of so‐called georeferenced natural history collections available to scientists (Randin, Engler, Pearman, Vittoz, & Guisan, 2009). Georeferencing is the process of interpreting the written description of site localities and verifying the associated geographic coordinates or assigning new coordinates (Rowe, 2005). Although no standard georeferencing process currently exists, many projects have developed individual guidelines (Chapman & Wieczorek, 2006). Examples of georeferencing practices and programs include the Mammal Networked Information System—MANIS guidelines (Wieczorek, Guo, & Hijmans, 2004; Wieczorek & Wieczorek, 2015), MapSteDI (Murphey et al., 2004), BioGeomancer (Chapman & Wieczorek, 2006), and GEOLocate (Rios & Bart, 2010). The two main branches of georeferencing methods are manual georeferencing and “Georeference Calculators.” Manual georeferencing requires the meticulous human interpretation of site descriptions and assigning coordinates using detailed topographic maps. This can take several minutes per sample and is increasingly taxing with large datasets. Georeference Calculators are computer algorithms designed to automate the tedious process of interpreting written site descriptions to estimate geographic coordinates and a degree of confidence (Wieczorek & Wieczorek, 2015). Many publications present SDM results, at varying spatial resolution, without explicitly stating how or if the data were georeferenced (Table 1).

**Table 1:** Examples of methods used to georeferenced species occurrence records as described in species distribution modeling (SDM) papers. Georeferencing practices are not standardized, and often the resolution of the resulting SDM is finer than the historical records used to train the model. Without accurately georeferenced presence points, it is impossible to create a credible SDM

Authors	Occurrence records source	SDM resolution	Georeference description
Jackson et al. (2015)	Field‐measured GPS localities and opportunistic citizen science sightings	100 m	For the field survey dataset, all locations were recorded with GPS. For citizen science program, summer observations filtered by location accuracy, retaining those with precise GPS or map coordinates (accurate to within 100 m)
DeChaine, Wendling, and Forester (2014)	Herbarium records	800 m	“Georeferenced” herbaria samples
Chardon, Cornwell, Flint, Flint, and Ackerly (2014)	Consortium of California Herbarium	800 m	Authors employed three criteria on herbarium records: (1) Omitted occurrences with GPS error larger than 1000 m; (2) If GPS error was not included in the occurrence file, only used specimens collected since the year 2000; (3) Omitted points that were clearly planted or outside of the species’ distribution
Lentz, Bye, and Sánchez‐Cordero (2008)	Herbarium records from the United States, United Kingdom, and Mexico	30 arc‐seconds (ca. 1 km^2^)	If the coordinates were not specified on herbarium records, the authors georeferenced using 1:100,000 topographic maps. Locality data were only used if the location of the collection could be accurately pinpointed
López‐Alvarez et al. (2015)	Herbarium records and field measured	30 arc‐seconds (ca. 1 km^2^)	Field collections and georeferenced collections
Smith and Donoghue (2010)	Labels on herbaria specimens, relevant herbaria databases, and other databases	30 arc‐seconds (ca. 1 km^2^)	No mention of georeferencing
Forester et al. (2013)	Online herbarium records	50 km	“georeferencing was evaluated for accuracy”

In this paper, we set out to answer the following question: What are the consequences of using occurrence data of varying levels of spatial accuracy to inform present and future SDMs for a high‐elevation plant? To address this question, first we outline a standardized method of georeferencing occurrence records specifically for building more useful SDMs, the Spatial Analysis Georeferencing Accuracy (SAGA) protocol. Next, to demonstrate the importance of a standardized process, we built current and future SDMs in MAXENT for the high‐elevation wildflower *Saxifraga austromontana* Wiegand (Saxifragaceae), using three sets of herbarium records, each georeferenced to a different level of spatial accuracy. Although we focus on a single plant species, the methods could be extended to any taxon with historical museum or herbarium occurrence records.

## 2 METHODS

### 2.1 Study system: *Saxifraga austromontana*

**Figure 1:**
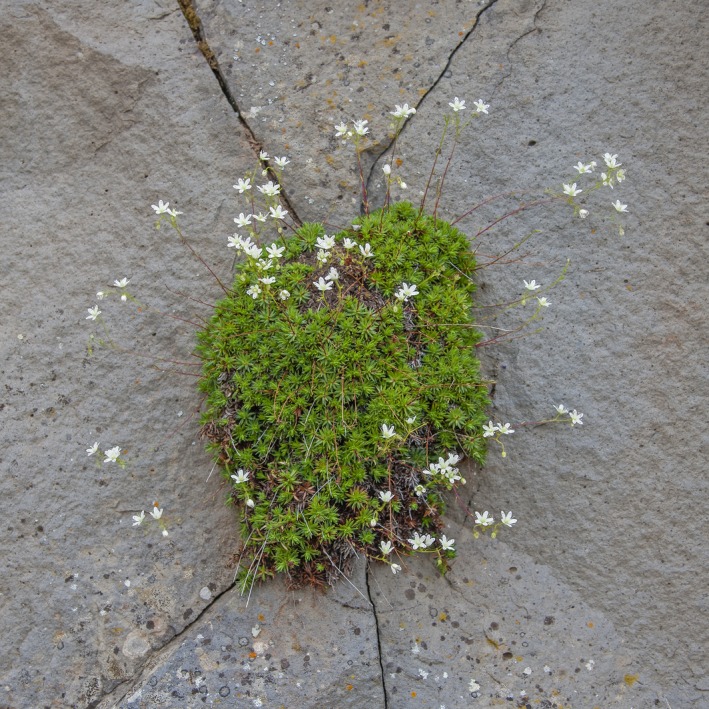
Saxifraga austromontana, the Prickly Saxifrage, is a charismatic wildflower endemic to upper elevations of the Rocky Mountain Floristic Region. The Latin name Saxifraga is known as rockfoils, sax meaning rock, and frage, to fracture. Here, it is shown growing from fissures in crags of the Rockies. Saxifraga austromontana grows perennially with low basal rosettes of spiny leaves and produces beautiful yet fragile flowers with cream colored petals dotted with red, orange, and yellow spots. This is an ideal case‐study species for investigating how various georeferencing methods affect SDM results because of its geographically large, but topographically limited, range and extensive herbarium records. (Photo credit, Dr. Eric DeChaine)

**Figure 2:**
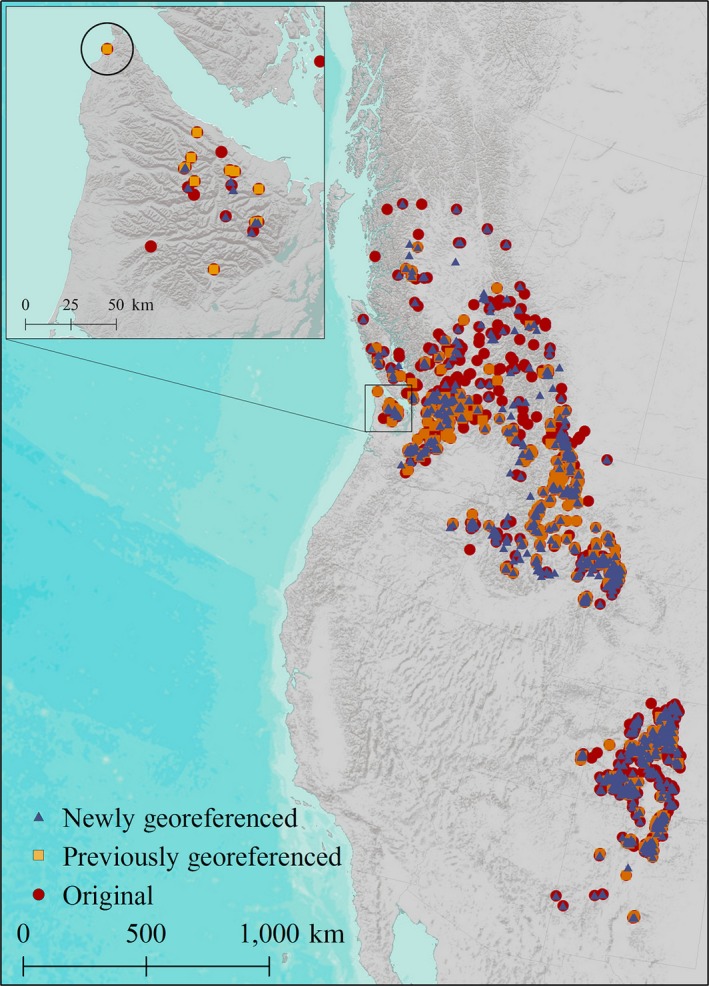
The distribution of Saxifraga austromontana for three categories of georeferenced historical herbarium records: Original data (O), Previously Georeferenced (PG), and Newly Georeferenced (NG). The circled point on inset map displays a species occurrence record on the coast of the Olympic Peninsula. The coordinate was incorrectly assigned using the georeference calculator: GeoLocate (WTU‐VP‐90424) and is included in both the O and PG dataset. Data are in a Lambert conformal conic equal area projection

*Saxifraga austromontana*, the Prickly Saxifrage, is an ideal case‐study species for investigating how various georeferencing methods affect SDM results because of its geographically large, but topographically limited, range and extensive herbarium records (Figure 1). First, this plant is endemic to, but widely distributed across, mountainous regions of western North America from 30 to 55 degrees’ latitude (Figure 2), where it inhabits a topographically complex region near tree line. Second, it has an extensive history of collections spanning over 200 years resulting in over 3,000 herbarium records available in online databases. The extensive collections of this species, and others in the genus with overlapping and extended ranges, limit the effect of sampling bias.

### 2.2 Historical herbaria record data

We compiled a complete “Original” (O) dataset of herbarium records for *S. austromontana*. In May 2015, we downloaded all search records for “*Saxifraga austromontana”* and its taxonomic synonym “*Saxifraga bronchialis”* from the Consortium of the Pacific Northwest Herbarium, Consortium of Intermountain Herbarium, Consortium of Rocky Mountain Herbarium, SEINet, and Canadensys. We included additional records from the Pacific Northwest Herbarium (WWB), University of Washington Herbarium (WTU), University of Oregon Herbarium (ORE), Mount Rainer National Park Herbarium (MORA), Royal BC Museum (V), University of British Columbia Herbarium (UBC), and the B.A. Bennett Herbarium (BABY).

The O dataset was edited to omit duplicate records and extreme outliers. Duplicate records across herbaria were found using accession numbers, GUID numbers, collector numbers, and site descriptions. Outliers were defined as occurrence records located very far outside of the known species range, such as records in the oceans, in the Great Plains, outside of North America, north of 55 degrees’ latitude (no confirmed records exist north of this latitude), and records in the state of Oregon outside of the Wallowa mountain range (the range of *S. vespertina*). Omission of outliers is common practice for building SDMs, yet not everyone goes beyond this step (Table 1). The O dataset includes 1,363 unique herbarium records (Figure 2).

The “Previously Georeferenced” (PG) dataset includes all records from the O dataset that explicitly state they have been georeferenced by other herbaria using a variety of methods. We omitted outliers and duplicates, as above, and removed records with coordinate uncertainty listed as >1 km. The final PG dataset includes 525 unique herbarium records (Figure 2).

The “Newly Georeferenced” (NG) dataset includes all historical herbarium records from the O dataset that we were able to manually georeference to a 1‐km or finer resolution. To conduct this manual georeferencing, we developed a novel method, the Spatial Analysis Georeferencing Accuracy (SAGA) protocol to standardize the process of georeferencing. We believe that the SAGA protocol is an improvement over other georeferencing practices in terms of both accuracy and straightforward implementation. This method is based on meticulously and manually georeferencing each herbarium record of interest and verifying written site descriptions using reliable external resources such as Google Earth, USGS Topographic Maps, and the Atlas of Canada to ensure accurate geographic coordinates. Each record must be reviewed, either through the online database it was downloaded from or by physically examining the herbarium specimen. All locations should be transformed into decimal degrees, with coordinates recorded relative to the WGS 1984 geodetic datum. Minimum spatial accuracy of each location following manual georeferencing should be recorded on an ordinal scale of 1–5 (Table 2) to allow for easy sorting and spatial analysis based on the spatial resolution of the occurrence data. We applied the SAGA protocol to the O dataset to create our NG dataset. The NG dataset only includes herbarium records with a confidence of 1–3 (Table 2) for a total of 1,104 unique historical herbarium records (Figure 2).

**Table 2:** Standardized confidence rankings for determining the spatial accuracy of species occurrence records using the Spatial Analysis Georeferencing Accuracy (SAGA) protocol. SAGA requires manual georeferencing of each occurrence record by interpreting the site location and verifying or assigning a location in the form of WGS 1984 geographic coordinates. The SAGA protocol uses an ordinal accuracy ranking of 1–5 to classify the spatial resolution of the occurrence data. Confidence ranks of 1–3 may be useful for constructing Species Distribution Models using 1‐km or coarser climate data. Ranks of 4 and 5 are not appropriate for spatial analysis and should be omitted

Confidence	GPS	Resolution (radius)	Description	Example accession nos
1	Required	1–30 m	Records with an accurate GPS reading, listed coordinate uncertainty, and a detailed written description that matches coordinates	WTU‐VP‐5827, RM‐VP‐740775
2	Sometimes	30–100 m	Records can be georeferenced to a fine resolution based on a detailed written description that can be verified, and in many cases a GPS reading. For example: summits of peaks, fire lookouts, intersections of creeks or trails	WTU‐VP‐185106, WTU‐VP‐90419
3	Sometimes	100–500 m	Record coordinates can be georeferenced to a moderate resolution based on a written description that can be verified. For example: small lakes, mountain passes, small named meadows	MONTU‐VP‐3979, WS‐VP‐101352
4	Often not	N/A	Record cannot be triangulated to a 1‐km grid. The site description may still be useful for collections, yet cannot be used in SDMs. For example: large lakes, entire mountains or peaks, ridgelines, trail names, well‐known geologic, or historic landmarks	MONTU‐VP‐27436, RM‐VP‐815188
5	Often not	N/A	Poor site description and coordinates cannot be verified. These data cannot be used accurately for SDMs and may not even be useful for collections. For example: town names, county names, state names, and mountain ranges	MONT‐VP‐50930, MONT‐VP‐50961

### 2.3 Species distribution models

We intentionally did not use all SDM approaches or an ensemble approach, but rather a widely used robust method to demonstrate the need for and utility of the standardized georeferencing protocol we present. We built SDMs using the MAXENT Software (Phillips, Anderson, & Schapire, 2006), one of the most, if not the most, widely used SDM platforms (Fourcade et al., 2014; Guillera‐Arroita et al., 2015; Merow, Smith, & Silander, 2013). MAXENT is built on machine learning and Bayesian statistics of maximum likelihood (Elith et al., 2011; Halvorsen, Mazzoni, Bryn, & Bakkestuen, 2015), and is especially popular because it outperforms other methods based on predictive accuracy and is user‐friendly (Merow et al., 2013).

The model inputs include a list of presence points, a set of environmental predictors (i.e., climate variables), and a defined background landscape. In contrast to a true presence–absence model, MAXENT estimates habitat suitability by contrasting environmental factors at presence points with thousands of randomly selected background points throughout the study region (Guillera‐Arroita et al., 2015). We followed MAXENT best practices (Merow et al., 2013) to build SDMs for *S. austromontana* using three categories of georeferenced data. Our models are intentionally simple to demonstrate the underlying importance of georeferencing.

### 2.4 Climate variables

We used monthly PRISM data (Daly et al., 2008) for the reference period (1961–1990) to define the bioclimatic envelope of *S. austromontana*. We felt that the (1961–1990) normal period, while a compromise, was representative of twentieth century conditions because (1) both the mean and median samples fell within the normal period, (2) the 30‐year climate normal allowed us to make comparisons with future projections, and (3) a 122‐year average across all sample dates was less meaningful given the amount that climate had changed. The PRISM methods utilize Digital Elevation Models to refine interpolation between climate stations by including factors such as location, elevation, and aspect (Daly et al., 2008). The climate data for this study were downscaled from 4 km^2^ grid cells to a resolution of 1 km^2^ and made available from ClimateWNA http://tinyurl.com/ClimateWNA (Hamann, Wang, Spittlehouse, & Murdock, 2013; Wang et al., 2012). We selected seven final variables for use in SDMs (Tables 3 and S3) using a multistep process. First variables were preselected from the complete list available for ecological relevance to our taxa and similar high‐elevation species (Körner, 1995, 2003). Next, we further reduced variables to eliminate highly correlated parameters (Pearson's *r* > |0.75|), Table 3. To decide between correlated variables, we relied on ecological relevance and informed judgment to select for a diverse suite of climate variables representing temperature, precipitation, heat moisture indexes, and more (Table 3). We also downscaled projected values of these variables for a 30‐year period centered on 2080. Future climate projections were obtained from ClimateWNA using an ensemble of 23 Atmosphere‐Ocean General Circulation Models (AOGCMs) of the Coupled Model Intercomparison Project phase 3 (CMIP3) under the A2 emission scenario, selected based on validation rank (Hamann et al., 2013).

**Table 3:** Climate variables selected for SDMs of *Saxifrage austromontana*, and percent contribution to MAXENT models for each of three levels of georeferencing: Newly Georeferenced (NG), Previously Georeferenced (PG), and Original (O). Top three contributing variables for each model are in bold. Climate data made available by ClimateNA for the reference period (1960–1990) and 2080 future projections based on an ensemble of 23 CMIP3 coupled atmosphere–ocean general circulation models (Hamann et al., 2013)

Variable	Description	NG	PG	O
AHM:	Annual heat moisture index, calculated as (MAT+10)/(MAP/1000)	4.6	8.9	.9
bFFP:	The Julian date on which the frost‐free period begins	**26.3**	**17.9**	**16.5**
cmiJJA:	Hogg's summer (Jun to Aug) climate moisture index	**21.2**	**26.5**	**35.4**
MCMT:	Mean temperature of the coldest month (°C)	10.3	7.8	**14.6**
MWMT:	Mean temperature of the warmest month (°C)	13	2.3	9.8
PAS:	Precipitation as snow (mm)	10.3	**23.9**	9.5
TD:	Difference between MCMT and MWMT, as a measure of continentality (°C)	**14.3**	12.7	13.1

### 2.5 Background selection

We limited the geographic background to locations within the likely dispersal range of *S. austromontana*. We trimmed the region extent for the reference period to the northern border of British Columbia, the southern border of the United States, and 150 km east of the Rocky Mountains. *Saxifraga austromontana* has been extensively collected across its range and is not found more than 150 km east of the Rocky Mountains crest, except for small isolated mountain ranges that we included in our extent. This area allowed us to include a potential northern range expansion, expected for cold‐adapted species (Forester et al., 2013).

### 2.6 Climate space analysis

To assess whether the occurrence records in each of our three georeferencing categories captured the same climatic envelopes, we quantitatively compared the climatic niche space for each dataset (O, PG, and NG) using Analyses of Variance (ANOVAs) and Principal Component Analysis (PCA). We ran one‐way ANOVAs to compare the variation between to the variation within each dataset for the values of seven climate variables extracted at each presence point. We used a Bonferroni correction to account for multiple testing, dividing the alpha of 0.05 by 3 for a final alpha of 0.017. We used an unrotated PCA to evaluate the climate space represented by the three levels of georeferenced data. We incorporated all climate variable values at all presence locations (O, PG, and NG combined) in our PCA and extracted the first two principal components. All statistics were run using R ver. 3.1.2 (R Core Team, 2015) and plotted using ggplot2 (Wickham, 2009).

### 2.7 MAXENT model settings

All SDMs were run using the version 3.3.3k of MAXENT (http://www.cs.princeton.edu/~schapire/maxent/). For ease of comparison among model outputs, all runs were computed with the default features (Linear, Quadratic, Product, Threshold, and Hinge), and a logistic output which results in a map of habitat suitability values ranging from 0 to 1 (Fourcade et al., 2014) per 1‐km grid cell, defined by the resolution of the input climate data. We set MAXENT to train each SDM to a random subsample of 75% of species presence points, with the remaining 25% of the data used for model evaluation. We increased the default maximum iterations to 5,000 and ran 20 replicates of each model.

### 2.8 Model evaluation

We evaluated the models using the area under the receiver operating curve (AUC) because it is a generally accepted and widely used metric for model evaluations (Merow et al., 2013). The AUC score is the probability that a randomly chosen presence point is ranked higher than a random background point, and is penalized for predictions outside of presence locations (Merow et al., 2013). A high AUC value (>0.8) indicates that models can properly distinguish between presences and random background samples. Although the AUC has been highly criticized as a metric of model performance (Lobo, Jiménez‐Valverde, & Real, 2008), there are few alternatives for presence‐only models (Merow et al., 2013).

To quantify the geographic differences between models created using occurrence records of varying accuracy, we used the 10% cumulative logistic threshold, which defines a binary response of suitable or nonsuitable habitat from a continuous output (Merow et al., 2013). Choosing biologically meaningful thresholds is challenging (Merow et al., 2013), yet this method can be used to easily compare the outputs of two or more models (Franklin et al., 2013). We compared area of suitable habitat for the reference and future predictions across the three georeferencing categories. Cartography and spatial comparisons were performed in ArcGIS 10.3.

## 3 RESULTS

### 3.1 Climate space analysis

**Figure 3:**
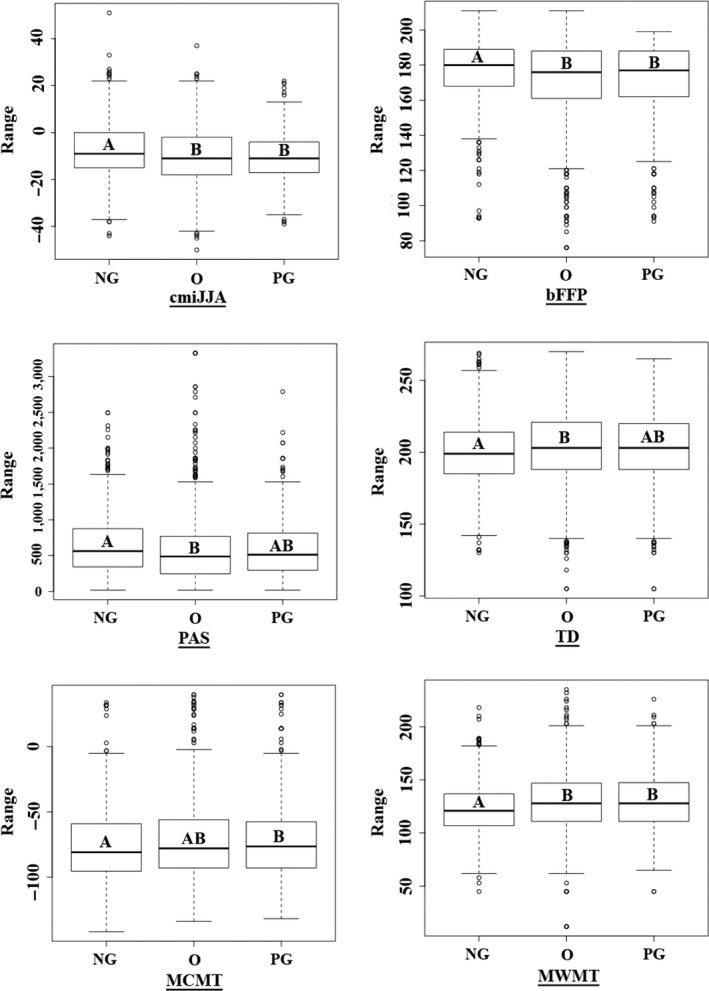
Range of values for seven climate variables extracted using each set of presence points for the three categories of georeferenced data: Newly Georeferenced (NG), Original (O), and Previously Georeferenced (PG). The plot displays the median, first and third quartiles, range, and extreme outliers. Different letters indicate a significant difference between datasets at a conservative alpha of 0.017, corrected with a Bonferroni

The NG dataset captures a significantly different range of environmental conditions than the other two datasets. The ANOVAs revealed that values extracted at each presence point in the O and NG datasets capture significantly different values for six of the seven climate variables (Figure 3 and Table S2). The PG and NG datasets capture significantly different values for five of seven climate variables. The O and the PG dataset do not significantly differ from each other in any of the climate variables. Effectively, O and PG capture the same climate envelope or the range of values within datasets are too large to detect a difference between groups.

**Figure 4:**
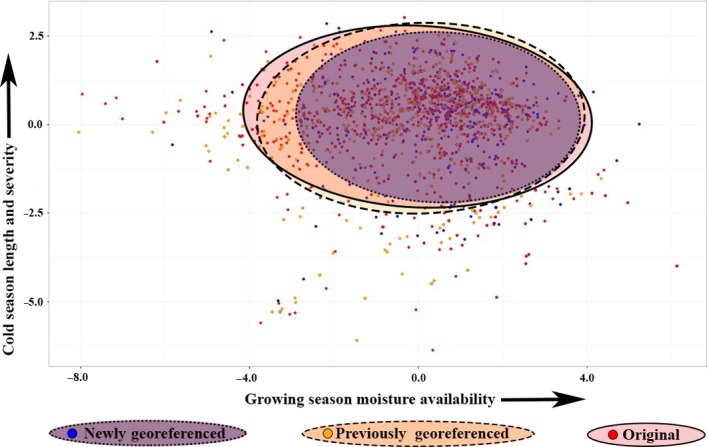
Principal Component Analysis (PCA) built on seven climate variables. Plots of niche space illustrate environmental differences and similarities among the three data sets: Newly Georeferenced (NG), Original (O), and Previously Georeferenced (PG). Principal component (PC) axes 1 and 2 account for 49.71% and 27.26% of the total variance. Ecologically, increasing PC1 can be interpreted as representing greater growing season moisture availability (more precipitation as snow (PAS), higher summer moisture index (cmiJJA), lower annual heat moisture index (AHM), and lower mean temperature of the warmest month (MWMT)). Higher values on PC2 represent increasing cold season length and severity (later start to the frost‐free period (bFFP), greater difference between summer and winter temperatures (TD), and colder winter temperatures (MCMT)). Cluster ellipses delineate 95% confidence intervals. For PCA loadings see Table S1

The differences between the climate envelopes captured by the three datasets are clearly visible when the presence points are plotted by their location in climate space, as represented by principal components (PC) axes 1 and 2. PC1 and PC2 extracted from all climate variables at all presence locations explain 49.71% and 27.26% of the total variance, respectively (Figure 4). Ecologically, increasing PC1 can be interpreted as representing greater growing season moisture availability (more precipitation as snow (PAS), higher summer moisture index (cmiJJA), lower annual heat moisture index (AHM), and lower mean temperature of the warmest month (MWMT)). Higher values on PC2 represent increasing cold season length and severity (later start to the frost‐free period (bFFP), greater difference between summer and winter temperatures (TD), and colder winter temperatures (MCMT)). The O dataset unequivocally captures the largest niche space, while the PG and NG are subsets of the O data. PG occupies most of the O dataset, whereas the NG dataset represents a much tighter ecological niche (Figure 4).

### 3.2 Species distribution models

**Figure 5:**
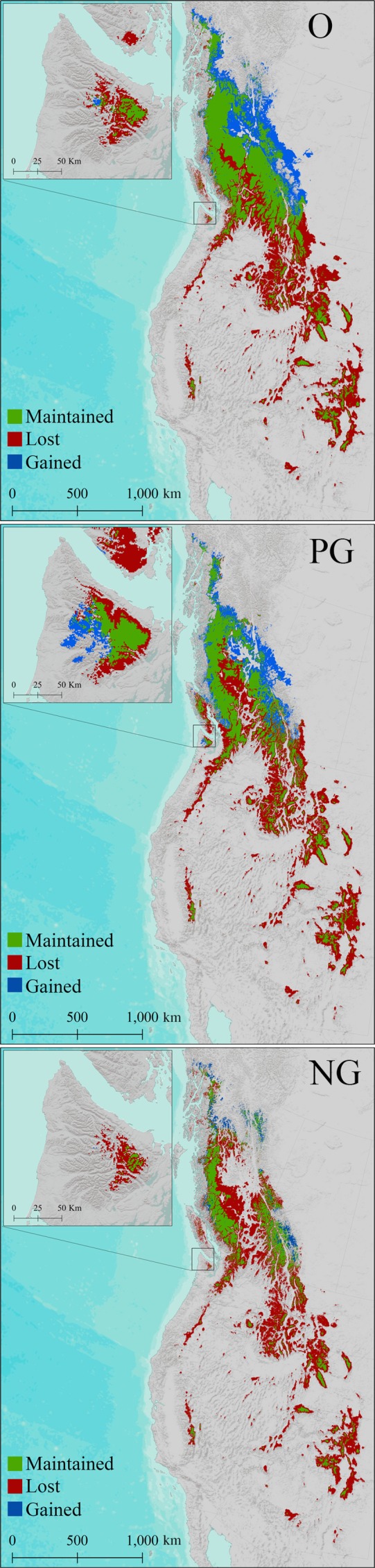
Species Distribution Model (SDM) of Saxifraga austromontana for the reference period (1960–1990) and 2080's under the A2 climate scenario for three categories of georeferenced data: Original (O), Previously Georeferenced (PG), and Newly Georeferenced (NG). Suitability is set at the 10‐percentile training presence logistic threshold. Projected for 2080, the O and PG models predict a relatively small reduction of 31.8% and 40.3%, respectively. The more NG model predicts a 65.7% reduction, more consistent with previous studies on alpine taxa (Table 4). The NG SDM does a good job of predicting present and future suitable habitat for Saxifraga austromontana. The O and PG SDMs overpredict suitable habitat outside of the known range of the target taxa, including locations on the coast of the Olympic Peninsula and Vancouver Island (see inset map). Inaccurate predictions of the O and PG dataset are exacerbated for future SDM outputs. Data are in a Lambert conformal conic equal area projection

**Figure 6:**
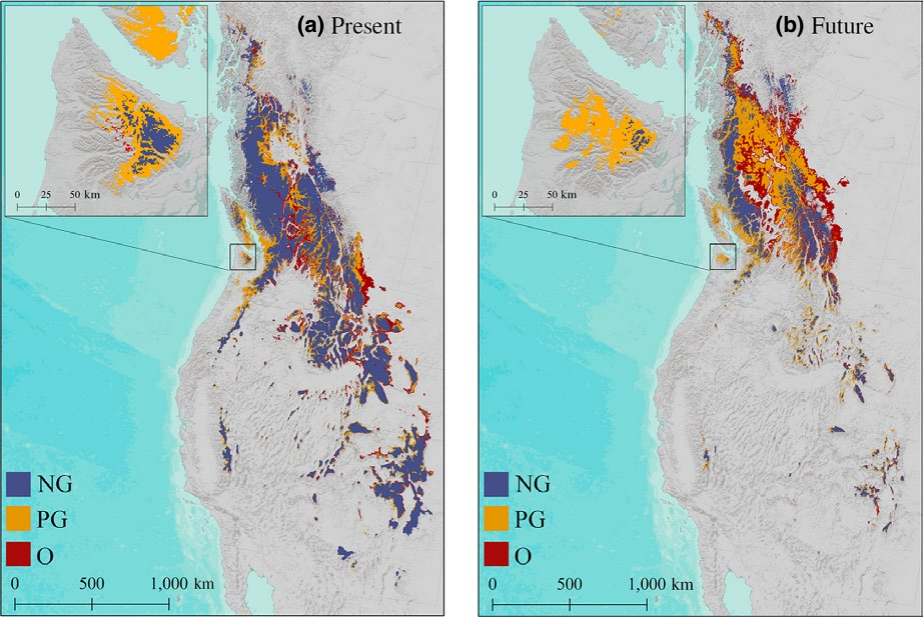
Species Distribution Models built using the three categories of georeferenced data (Original (O), Previously Georeferenced (PG), and Newly Georeferenced (NG)) result in notably different areas of suitable habitat for the (A) reference period (1960–1990) and (B) 2080 under the A2 emission scenario. SDM results based on the NG dataset are overlaid on top of SDM results using the O and PG datasets to visualize the differences in predicted niche space. The O and PG datasets greatly overpredict suitable habitat for the target taxa into regions it is known to be absent, including the coast of the Olympic Peninsula and Vancouver Island. This is due to the inclusion of inaccurate presence points such as WTU‐VP‐90424, displayed in Fig. 2. Data are in a Lambert conformal conic equal area projection

All MAXENT models were statistically valid (AUC > 0.88); however, the models predicted very different areas of suitable habitat, especially for future scenarios (Figure 5 and Figure 6, Table 4). The SDMs for the reference period (1960–1990) constructed using NG data resulted in the smallest area of suitable habitat, equivalent to 84.3% of the area of the SDM constructed using PG data and 71.5% of the area of the SDM constructed using O data (Figure 6a). The 2080 SDM results for the three categories of georeferenced data differed even more drastically (Figures 5 and 6b, Table 4). The SDM constructed using NG data predicted the smallest area of suitable habitat, equivalent to 50% of the area of the SDM trained using PG data and 37.1% of the area of the SDM trained using O data. The future SDM using NG data estimated the greatest loss and smallest gain in suitable habitat by 2080. The models also differed in the relative contribution of each climate variable (Table 3). The larger geographic ranges predicted by the O and PG models are a natural outcome of the larger climatic ranges captured by those datasets. Varying accuracy of occurrence records results in considerable differences in how SDMs project the location of this species in both climatic space and geographic space.

**Table 4:** The results of MAXENT models for *Saxifraga austromontana* trained on presence points from three levels of georeferenced data: Original (O), Previously Georeferenced (PG), and Newly Georeferenced (NG) with the SAGA protocol. All models were run with the same features and climate covariates. The total percent reduction in the future area of suitable habitat relative to the reference period is presented in bold. The O and PG models overpredict present suitable habitat with respect to the more accurate NG model, and the shortcomings of the O and PG models are exacerbated for the future projection. All models have high validation statistics using the area under the receiver operating curve (AUC) value, providing additional evidence to the argument that AUC scores are not a reliable metric for model accuracy

Dataset	Original	Previously georeferenced	Newly georeferenced
AUC	0.888	0.914	0.914
Reference Period (km^2^)	913,695	775,270	653,898
Future 2080s (km^2^)	623,044	462,658	231,376
Lost (km^2^)	477,235	447,353	461,758
Gained (km^2^)	186,584	134,741	39,236
Maintained (km^2^)	436,460	327,917	192,140
**Total Reduction (%)**	**31.8**	**40.3**	**65.7**

## 4 DISCUSSION

A standardized process is needed to ensure consistent spatial accuracy of species occurrence records for use in SDMs. We employed the most commonly used SDM tool, MAXENT, and our findings are broadly applicable to correlative SDMs. The method used to georeference museum records greatly influences the spatial accuracy of those points, and thus the results of SDMs. Georeferencing manually increased the number of valid presence points available, with the NG model incorporating more than twice the number of points compared to the PG model (1,104 vs. 525). A standardized georeferencing protocol can thus increase both the accuracy and number of available species occurrence records, simultaneously expanding the geographic coverage of those records and refining the climatic envelope they capture.

Although all three of our SDMs had high validation statistics (AUC > 0.88), the SDMs constructed using the O and PG datasets captured significantly different climatic envelopes for *S. austromontana* than the SDM trained using NG data. The O and PG datasets include many points that are clearly beyond the known range of *S. austromontana*. Although these points are outside the species’ range, at first glance they may not be considered extreme outliers, and would likely be used in an analysis that does not preprocess with manual georeferencing. For example, on the Olympic Peninsula of Washington State, both the O and PG datasets include a point on the shore of Lake Crescent near the town of Piedmont at an elevation of 198 m (WS‐VP‐70650), where the site description states the sample was collected on Mt. Storm King at an elevation between 1,311 and 1,829 m. The incorrectly estimated point is over 6 km off and captures a completely different elevation and climate space than the actual collection site. Another example on the Olympic Peninsula is a point <500 m from the western coast at an elevation of 104 m (WTU‐VP‐90424), included in both the O and PG datasets (Figure 2). This point was estimated, quite inaccurately, by the WTU herbarium using the GeoLocate calculator. The Pacific Northwest Herbarium (WWB), which has conducted extensive surveys on the Olympic Peninsula and works closely with Olympic National Park, has not recorded any *S. austromontana* in coastal or low‐elevation sites.

Numerous other inaccurate records were corrected using our manual georeferencing protocol. Common errors were coordinates taken at the trailhead, or in one instance the latrine, often with a GPS, rather than the actual collection site. Consequently, we feel confident stating that the NG dataset captured a more accurate representation of the species’ occupied climate space. Thus, the NG dataset provides a more realistic estimate of the climatic conditions in which *S. austromontana* exists: a cooler, wetter environment with a shorter‐growing season (Figure 4). Those conditions are more consistent with the known habitat of this high‐elevation plant, compared to the climate envelopes of the O and PG datasets. The models run using the O and PG datasets did not capture significantly different climate space compared with each other (Figure 3). This indicates that the PG dataset is not much better than the O dataset at defining the specific niche of *S. austromontana*.

The differences in climate space among our models led to drastically different SDM outputs and strikingly different predictions of current and future ranges. Using the 10% cumulative logistic threshold to define a binary response of suitable or nonsuitable habitat, the O and PG models resulted in suitable habitat covering geographic areas 1.4 and 1.2 times larger than the NG dataset for the reference period. Erroneously placed presence locations, such as WTU‐VP‐90424 circled in Figure 2, create a broader envelope for the target taxon. For example, the O and PG datasets show suitability across most of the Olympic Peninsula and southern Vancouver Island including coastal regions that have been well‐documented botanically and do not currently contain *S. austromontana*. Interestingly, the O dataset is more accurate than the PG in predicting the range on the Olympic Peninsula and Vancouver Island, probably because it includes more reference points. The NG SDM captures a much more accurate and tighter representation of the current range of *S. austromontana,* which is abundant primarily in the northeastern arc of basaltic peaks in the Olympics (Figures 5 and 6a).

It is important to note that all models (O, PG, and NG) predict habitat outside of the known range of *S. austromontana,* including the Sierra Nevada, Uinta, and Wind River ranges. These regions are within the climate envelope of the species, yet for alternative reasons (e.g., dispersal and competition dynamics), the species is not known to occur there, despite extensive botanical surveys. Overall, the O and PG datasets create SDMs that appear to overpredict suitable habitat in comparison with the NG data based on our current understanding of this species’ ecology. These results clearly demonstrate the shortcomings of unvalidated presence datasets for use in SDM construction.

Differences in predicted area of suitable habitat among the O, PG, and NG datasets are even more pronounced for future predictions. Our results are based on relatively simple model settings and should be treated as a visualization of the effects of georeferencing methods and coordinate accuracy on extrapolated future ranges, rather than as precise future predictions. The NG SDM estimates a 65.7% reduction in suitable habitat by 2080, while the SDMs constructed using the other datasets estimate a 32%–40% reduction by 2080, under the A2 emission scenario. The NG models are more consistent with other studies on alpine taxa that forecast a 40%–80% reduction in suitable habitat by the end of the century (Dirnböck, Essl, & Rabitsch, 2011; Dullinger et al., 2012; Forester et al., 2013). Further, the NG model predicts a relatively small gain in habitat by 2080, equivalent to 21%–29% of the area of gain predicted by the other two models, explained by limited upslope habitat for alpine taxa. Such underprediction of future range loss is worrying for any species, but especially for high‐elevation species, which are disproportionately affected by climate change (Gottfried et al., 2012) and often have little room for upward range expansion (Jackson, Gergel, & Martin, 2015).

Relying on potentially inaccurate presence records when modeling species’ ranges could lead to serious overestimation of the area in which these species can persist, misleading conservation and management efforts. SDMs can be developed to their full potential only when they are trained using many high‐precision occurrence records for a species (Randin et al., 2009). Our results demonstrate that there is no alternative for highly accurate presence data that have been meticulously georeferenced by a human, not a machine. Many SDMs are built using historical museum or herbarium records. In fact, for many taxa, these datasets are the only available records of their distribution. We found that geographic coordinates published on reputable herbaria sites often do not match the site description. These coordinates may have been recorded inaccurately by the collector, estimated by the collector using a coarse‐scale topographic map, recorded in a different geographic coordinate system than present systems (i.e., using NAD27 vs. WGS84 as the geodetic datum), georeferenced incorrectly by a curator, or estimated using a Georeference Calculator.

We have found the results of Georeference Calculators (Wieczorek & Wieczorek, 2015; GeoLocate 2016) to be frequently misleading, often adding an element of sampling bias by assigning coordinates for collections taken in the mountains to the nearest town. For example, we tested the utility of the GeoLocate Web Application Standard Client to assign a coordinate to the locality string “West Ute Lake, Weminuche Wilderness,” Country: “United States of America,” State: “Colorado,” County: “Hillsdale.” The program assigned a coordinate with an uncertainty code of 301 m to 37.466673, −106.978932, which is more 30 miles southeast of the true location of West Ute Lake. These calculators are popular because they are easy to use and allow for batch processing of CSV files with many listed localities, but the spatial accuracy of these outputs is questionable.

## 5 CONCLUSION AND FUTURE EFFORTS

Understanding the present and future distributions of species is critical for applications in conservation, ecology, biogeography, phylogenetic analysis, phenology, landscape ecology, and beyond (Davis et al., 2015; Fois, Fenu, Lombraña, Cogoni, & Bacchetta, 2015; Forester et al., 2013; Lenoir, Gégout, Marquet, De Ruffray, & Brisse, 2008; Newbold, 2010). SDMs, especially those implemented in MAXENT, are the most common tools used to determine habitat suitability. As these tools become more and more popular and public access to species occurrence data increases, it is paramount to remember that convincing SDMs can be produced from dubious data (Lozier et al., 2009). Museum and herbaria databases are invaluable archives of occurrence information, yet must be used with caution, especially when applied to spatial analyses. Our results indicate that SDMs built using low‐accuracy location data capture a significantly broader climate envelope, predict a more widespread spatial distribution, and predict less loss under climate change scenarios than SDMs trained on accurate collection records. Conservation and management decisions could vary considerably depending on which model's output they were based on.

This study highlights the importance of meticulously georeferencing all records manually before use in SDMs and reveals the need for a standardized protocol such as SAGA, as varying levels of georeferencing result in significantly different models of habitat suitability for the same species. The tradeoff of manual georeferencing is the time it takes to analyze each record. As datasets increase in size, the feasibility of georeferencing each record becomes increasingly daunting. Batch georeferencing calculators may be desirable for large datasets, but reliable technology is not yet available. As the resolution of historical and projected climate data increases, more advanced and accurate SDMs become possible, but only if species occurrence records are also available at an increasingly fine scale. Field collectors must record accurate coordinates, GPS uncertainty, and detailed site descriptions, assuming use in future spatial analyses. Curators of databases must only make available accurately georeferenced occurrence records, or explicitly state otherwise. Lastly, end users must suspect occurrence records to be inaccurate and georeference before performing spatial analyses using a protocol such as SAGA. All parties should share the improved data, ultimately improving publicly available datasets and resulting science.

## CONFLICT OF INTEREST

None declared.

## AUTHOR CONTRIBUTION

Trevor Bloom was responsible for project development as part of his MS thesis at Western Washington University. He conducted the data collection and led the analyses, interpretation, and writing. Aquila Flower advised the spatial analyses and interpretation of the results; she also greatly assisted in the writing and editing of the publication. Eric DeChaine served as Trevor Bloom's thesis adviser and was the overall project supervisor. He assisted in the formulation and development of the study, specimen acquisition, data interpretation, and writing.
