# Supplementary figures and images for: Why georeferencing matters: Introducing a practical protocol to prepare species occurrence records for spatial analysis
Source: Ecol Evol. 2017 Dec 6;8(1):765–77. doi: 10.1002/ece3.3516 (PMC5756859; doi:10.1002/ece3.3516)

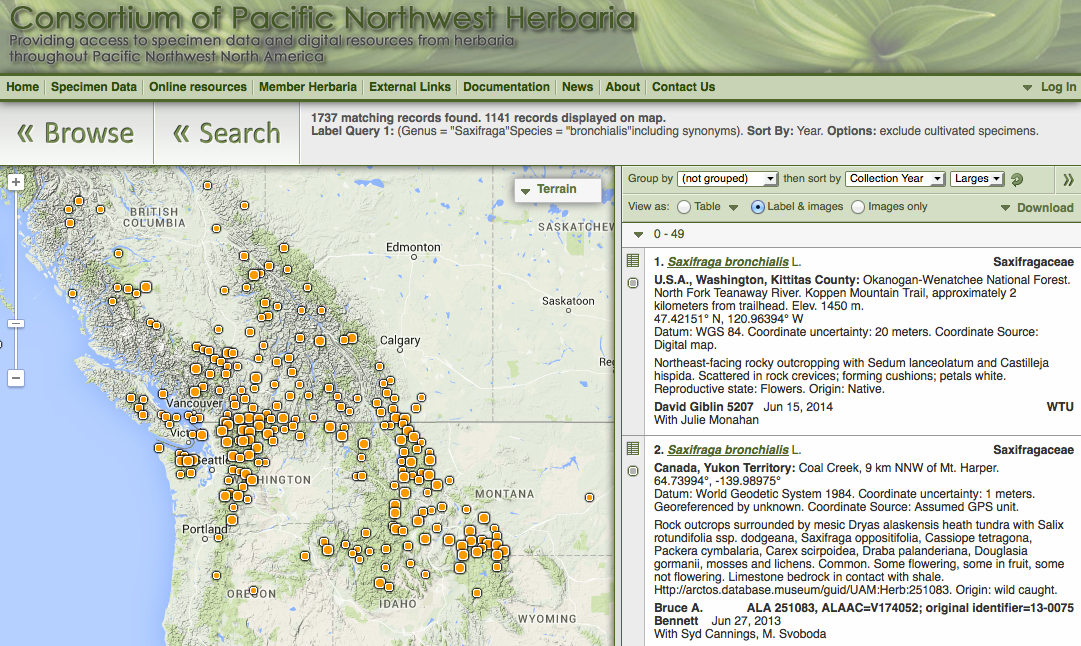

Supplement: Supplementary file 1 [file ECE3-8-765-s001.tiff]
