# Supplementary material for: Why georeferencing matters: Introducing a practical protocol to prepare species occurrence records for spatial analysis
Source: Ecol Evol. 2017 Dec 6;8(1):765–77. doi: 10.1002/ece3.3516 (PMC5756859; doi:10.1002/ece3.3516)
Supplement: Supplementary file 2 [file ECE3-8-765-s002.docx]

**(A) SUPPLEMENTARY MATERIAL**

**Table S1:** PCA loadings for niche space analysis of *Saxifraga austromontana*. Principal component (PC) axes 1 and 2 account for 49.71% and 27.26% of the total variance, respectively. The top contributing variables for PC1 and PC2 are in bold.

| **Variable:** | **PC1** | **PC2** | **PC3** | **PC4** | **PC5** | **PC6** | **PC7** |
| --- | --- | --- | --- | --- | --- | --- | --- |
| AHM | **-0.47** | 0.15 | 0.19 | 0.19 | 0.83 | 0.03 | 0.00 |
| bFFP | 0.28 | **0.45** | 0.52 | -0.40 | 0.07 | -0.53 | 0.00 |
| cmiJJA | **0.44** | -0.22 | -0.24 | 0.56 | 0.23 | -0.58 | 0.00 |
| MCMT | -0.27 | **-0.59** | 0.19 | -0.23 | -0.03 | -0.30 | -0.63 |
| TD | -0.18 | **0.57** | -0.57 | -0.02 | -0.06 | -0.16 | -0.54 |
| PAS | **0.41** | -0.19 | -0.40 | -0.60 | 0.49 | 0.17 | 0.00 |
| MWMT | **-0.48** | -0.12 | -0.34 | -0.28 | -0.09 | -0.49 | 0.56 |
| Standard deviation | 1.87 | 1.38 | 0.91 | 0.64 | 0.48 | 0.38 | 0.00 |
| Proportion of Variance | 0.50 | 0.27 | 0.12 | 0.06 | 0.03 | 0.02 | 0.00 |
| Cumulative Proportion | 0.50 | 0.77 | 0.89 | 0.95 | 0.98 | 1.00 | 1.00 |

**Table S2**: Results from ANOVA analyses comparing the variation between to the variation within each dataset for the values of seven climate variables extracted at each presence point. The Newly Georeferenced (NG) dataset differs significantly from the Original (O) and Previously Georeferenced (PG) dataset for 6 of 7 and 5 of 7 climatic variables, respectively. The O and PG datasets do not significantly differ for any one variable. We used a Bonferroni correction to account for multiple comparisons across the three datasets. Significance code: p < 0.017*.

| **Variable** | **NG vs. O** | **NG vs. PG** | **PG vs. O** |
| --- | --- | --- | --- |
| AHM | 1.49e-10* | 0.005* | 0.02 |
| bFFP | 1.23e-08* | 2.57e-05* | 0.85 |
| cmiJJA | 8.76e-07* | 1.4e-08* | 0.094 |
| PAS | 9.49e-05* | .056 | 0.24 |
| MCMT | 0.022 | .0055* | 0.28 |
| MWMT | 8.5e-10* | 2.3e-07* | 0.81 |
| TD | 1.9e-03* | .059 | 0.33 |

**Table S3:** All bioclimatic variables made available by ClimateNA <http://ualberta.ca/~ahamann/data/climatena.html>.

| **23 Bioclimatic variables:** |  |
| --- | --- |
| MAT: | mean annual temperature (°C) |
| MCMT: | mean temperature of the coldest month (°C) |
| MWMT: | mean temperature of the warmest month (°C) |
| TD: | difference between MCMT and MWMT, as a measure of continentality (°C) |
| MAP: | mean annual precipitation (mm) |
| MSP: | mean summer (May to Sep) precipitation (mm) |
| AHM: | annual heat moisture index, calculated as (MAT+10)/(MAP/1000) |
| SHM: | summer heat moisture index, calculated as MWMT/(MSP/1000) |
| DD.0: | degree-days below 0°C (chilling degree days) |
| DD.5: | degree-days above 5°C (growing degree days) |
| NFFD: | the number of frost-free days |
| bFFP: | the Julian date on which the frost-free period begins |
| eFFP: | the Julian date on which the frost-free period ends |
| PAS: | precipitation as snow (mm) |
| EMT: | extreme minimum temperature over 30 years (°C) |
| Eref: | Hargreave's reference evaporation |
| CMD: | Hargreave's climatic moisture index |
| CMI: | Hogg's climate moisture index |
| cmiJJA: | Hogg's summer (Jun to Aug) climate moisture index |
| Tave_wt: | winter (Dec to Feb) mean temperature (°C) |
| Tave_sm: | summer (Jun to Aug) mean temperature (°C) |
| PPT_wt: | winter (Dec to Feb) precipitation (mm) |
| PPT_sm: | summer (Jun to Aug) precipitation (mm) |
